# Pathogenic *p62/SQSTM1* mutations impair energy metabolism through limitation of mitochondrial substrates

**DOI:** 10.1038/s41598-017-01678-4

**Published:** 2017-05-10

**Authors:** Fernando Bartolome, Noemi Esteras, Angeles Martin-Requero, Claire Boutoleau-Bretonniere, Martine Vercelletto, Audrey Gabelle, Isabelle Le Ber, Tadashi Honda, Albena T. Dinkova-Kostova, John Hardy, Eva Carro, Andrey Y. Abramov

**Affiliations:** 1 Neurodegenerative Disorders group, Instituto de Investigacion Hospital 12 de Octubre (i+12), Av Cordoba, Madrid 28041 Spain; 2 Biomedical Research Networking Centre on Neurodegenerative Diseases (CIBERNED), Madrid, Spain; 3 Department of Molecular Neuroscience, UCL Institute of Neurology Queen Square, London, WC1N 3BG UK; 4 Department of Cellular and Molecular Medicine, Centro de Investigaciones Biológicas (CSIC), Ramiro de Maeztu 9, Madrid, 28040 Spain; 5 Biomedical Research Networking Centre on Rare Diseases (CIBERER), Madrid, Spain; 6 Laboratoire d’études des mécanismes cognitifs, EA 3082, Université Lyon 2, Bron, F-69500 France; 7 CHU Nantes, Centre de Mémoire et de Ressource et Recherche (CM2R), Nantes, France; 8 Inserm, CIC 04, Nantes, France; 9 Memory Research and Resources Center, Department of Neurology, Montpellier University Hospital, Montpellier, France; 10 CNR-MAJ, AP-HP, Hôpital de la Pitié-Salpêtrière, Paris, France; 11 ICM, Inserm U1127, CNRS UMR 7225, Sorbonne Universités, UPMC-P6 UMR S 1127 - Hôpital Pitié-Salpêtrière, Paris, France; 12 Department of Chemistry and Institute of Chemical Biology & Drug Discovery Stony Brook University Stony Brook, New York, 11794 USA; 13 Division of Cancer Research, School of Medicine University of Dundee, Dundee, DD1 9SY UK; 14 Reta Lilla Weston Laboratories, London, WC1N 3BG UK

## Abstract

Abnormal mitochondrial function has been found in patients with frontotemporal dementia (FTD) and amyotrophic lateral sclerosis (ALS). Mutations in the *p62* gene (also known as *SQSTM1*) which encodes the p62 protein have been reported in both disorders supporting the idea of an ALS/FTD continuum. In this work the role of p62 in energy metabolism was studied in fibroblasts from FTD patients carrying two independent pathogenic mutations in the p62 gene, and in a p62-knock-down (*p62* KD) human dopaminergic neuroblastoma cell line (SH-SY5Y). We found that p62 deficiency is associated with inhibited complex I mitochondrial respiration due to lack of NADH for the electron transport chain. This deficiency was also associated with increased levels of NADPH reflecting a higher activation of pentose phosphate pathway as this is accompanied with higher cytosolic reduced glutathione (GSH) levels. Complex I inhibition resulted in lower mitochondrial membrane potential and higher cytosolic ROS production. Pharmacological activation of transcription factor Nrf2 increased mitochondrial NADH levels and restored mitochondrial membrane potential in p62-deficient cells. Our results suggest that the phenotype is caused by a loss-of-function effect, because similar alterations were found both in the mutant fibroblasts and the p62 KD model. These findings highlight the implication of energy metabolism in pathophysiological events associated with p62 deficiency.

## Introduction

p62, also known as sequestosome 1, is a scaffold or an adaptor protein involved in multiple cellular activities and is encoded by the *p62* gene (also known as *SQSTM1*). Mutations in the *p62* gene have been found to cause both amyotrophic lateral sclerosis (ALS) and frontotemporal dementia (FTD). FTD and ALS are sometimes associated in patients or within families showing ALS and FTD as a pathological continuum (ALS/FTD) as they share common pathological features^1^. Supporting this idea, mutations in the same disease-causing genes in both disorders have been reported. These include *VCP*^2^, *p62*^3^, *OPTN*^4^, *UBQLN2*^5^ and especially the hexanucleotide repeat expansion in *C9orf72*^6^. It is worth mentioning that mutations in *OPTN*, *VCP* and *p62* also cause Paget disease of the bone (PDB)^1^. p62 has been related to neurodegenerative phenotypes and it has been linked to the ubiquitin-proteasome system and autophagy, main protein degradation mechanisms in cells. Protein aggregates containing p62 have been also found in many disorders, including ALS and FTD^7,8^, in some cases colocalizing with the transactive response DNA-binding protein 43 (TDP-43)^9^ and also in ubiquitinated inclusions along with FUS protein and TDP43^10^.

The link between p62 and neurodegeneration has been further investigated using animal and cell models. Most of them have focused on autophagy, the ubiquitin-proteasome degradation pathway and particularly, the mitochondrial quality control process known as mitophagy^11–15^. Studies with cell models showed the implication of p62 in mitophagy which is disrupted in some forms of Parkinson’s disease^11,15^. The silencing of the *p62* orthologue in drosophila *ref*(2)*P*, resulted in mitochondrial dysfunction, and mitochondrial DNA accumulation and this was linked to the observed locomotor deficits in the flies^12,13^. In 2014, Seibenhener and colleagues further showed the link between p62 and mitochondrial protein turnover using p62 silenced mouse embryonic fibroblasts (MEFs)^14^. Mitochondria are essential organelles especially for neurons as they are the main source of ATP due to limited glycolysis. The p62 knockout mouse model as well as p62 silenced MEFs showed impaired mitochondrial function resulting in reduced ATP production^14,16,17^.

Accumulating evidence from recent studies suggests that mitochondrial dysfunction plays a significant role in both the FTD and ALS etiopathogenesis^18–21^. This has been demonstrated using different animal and cell models. In particular, we recently demonstrated that mutations in *VCP* cause mitochondrial uncoupling leading to decreased mitochondrial membrane potential and a significant reduction of cellular ATP production highlighting the pathophysiological events that may occur in FTD and ALS^22^. In the present study we aimed to analyze mitochondrial function and pathophysiology using fibroblasts from FTD diagnosed patients carrying two independent *p62* mutations and the p62-knock-down (*p62 KD*) human dopaminergic neuroblastoma cell line (SH-SY5Y) model. We found that p62 deficiency induces inhibition of mitochondrial respiration due to a lack of substrate delivery. This inhibition results in higher cytosolic ROS production inducing the cells to increase the pentose phosphate pathway activity (PPP) in order to enhance the reduced glutathione (GSH) levels. We also demonstrated that the p62 mutations cause a loss-of-function effect as the *p62 KD* shows the same phenotype as the p62 mutations from patients.

## Results

### p62 deficiency is associated with decreased mitochondrial membrane potential (ΔΨ_m_)

**Figure 1:**
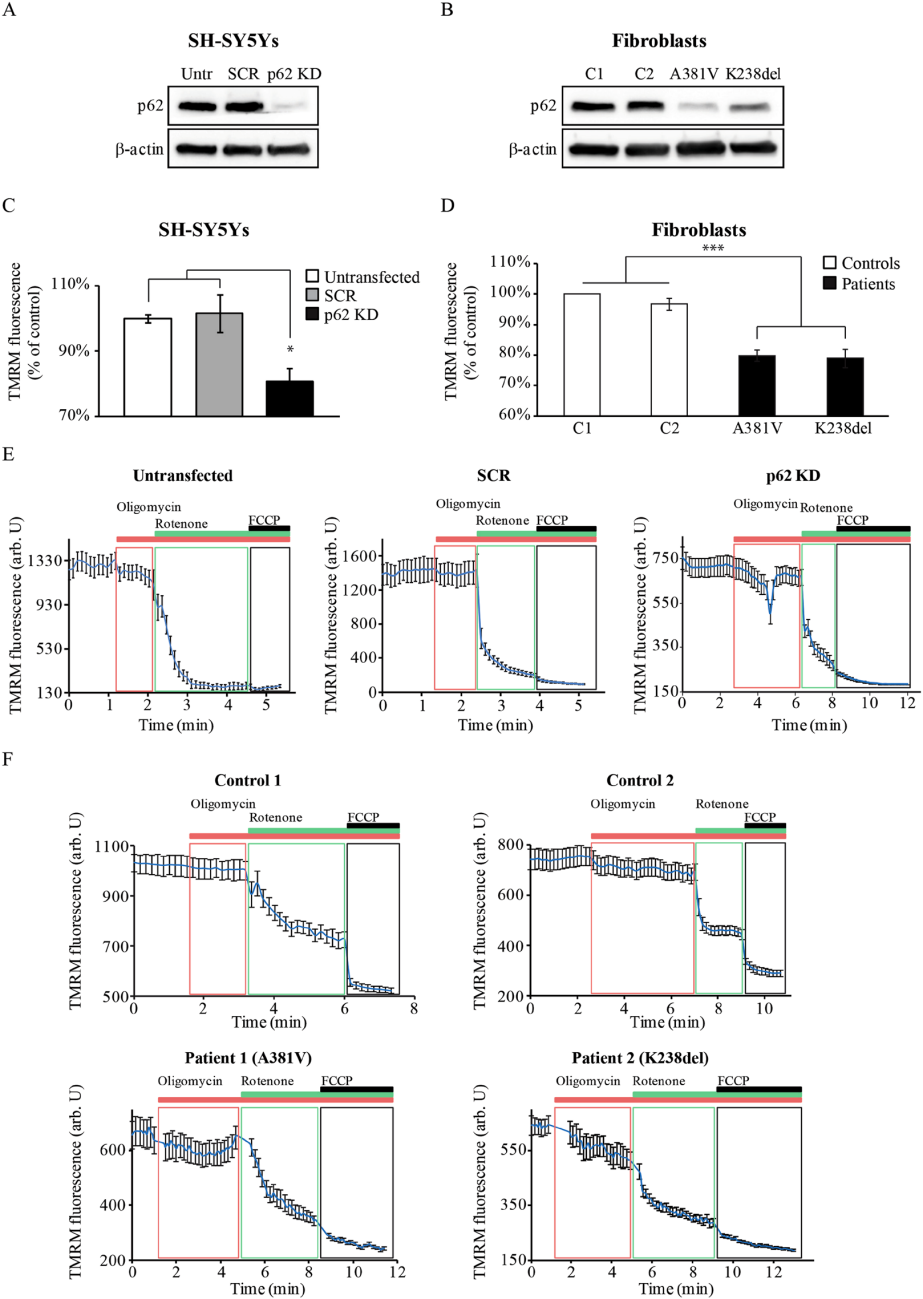
p62 deficiency induces mitochondrial depolarisation. (**A,B**) Immunoblotting showing the p62 protein levels corresponding to whole cell lysates from untransfected (Untr) and transiently transfected (either with SCR or p62 siRNA) SH-SY5Y cells (**A**) or fibroblasts from patients carrying p62 mutations and aged-matched controls C1 and C2 (**B**). In both A and B, β-actin was used as loading control. (**C,D**) Mitochondrial membrane potential (ΔΨ_m_) was estimated by live cell imaging in untransfected, scramble siRNA KD (SCR) and *p62 KD* SH-SY5Y cells (**C**) and in fibroblasts from patients carrying A381V and K238del pathogenic mutations in *p62* compared to fibroblasts from two healthy donors (C1, C2) (**D**) using TMRM in a redistribution mode (40 nM). Data were normalised to control SCR cells (**C**) and control fibroblasts (**D**) and are represented as mean ± SEM from at least three independent experiments. (**E,F**) Representative TMRM traces from untransfected, SCR and *p62 KD* SH-SY5Y cells (**E**) and fibroblasts from control donors (**F, upper panels**) and fibroblasts from patients carrying the *p62* pathogenic mutations mentioned above (**F, bottom panels**). The charts show responses to oligomycin (2 μg/mL), rotenone (5 μM) and FCCP (1 μM). In all cases *indicates p < 0.05 and ***indicates p < 0.001 compared with the values of control cells.

Mitochondrial health and function are reflected in the mitochondrial membrane potential (ΔΨ_m_). The implication of p62 in the mitochondrial function was assessed by transient silencing of the *p62* gene in the SH-SY5Y human neuroblastoma cell line using siRNA (*p62 KD*) (Fig. 1A). The ΔΨ_m_ was then measured using tetramethyl-rhodamine methylester (TMRM) as a fluorescent indicator of ΔΨ_m_ in the *p62 KD* cells and in mutant fibroblasts from patients carrying independent *p62* mutations (patient 1 = A381V; patient 2 = K238del; for donor’s details see Supplementary Fig. 1 and Table 1). Interestingly, the p62 protein levels in the mutant fibroblasts were reduced in both carriers but more remarkably in patient 1 carrying the A381V mutation (Fig. 1B). A significant decrease in ΔΨ_m_ was observed in *p62 KD* SH-SY5Y cells reducing the TMRM signal to 81 ± 4% (n = 4) compared to either untransfected (100 ± 1%, n = 4) or cells transfected with scrambled (SCR) siRNA control (102 ± 6%, n = 4) (Fig. 1C). Equivalent effects on the ΔΨ_m_ were observed in the mutant fibroblasts when compared to age-matched controls (Fig. 1D). The TMRM fluorescence signal was significantly reduced in fibroblasts with the pathogenic *p62* mutations, indicating that basal ΔΨ_m_ is also reduced in these cells (control 1 = 100 ± 1%, n > 10; control 2 = 97 ± 2%, n = 5; patient 1 = 80 ± 3%, n > 10; patient 2 = 79 ± 3%, n > 10) compared to control fibroblasts (Fig. 1D).

**Table 1:** Donors’ information.

	Sex	Current Age	Age of onset	Family history	Clinical features	Clinical diagnosis	*p62* mutation
Control 1	M	81	—	—	unaffected	—	—
Control 2	F	52	—	—	unaffected	—	—
Patient 1	F	54	48	yes	Behavioural disturbances (Le Ber *et al*. 2013)	bvFTD	A381V
Patient 2	F	82	75	yes	Speech apraxia (Boutoleau-Bretonniere *et al*. 2015)	nvfFTLD	K238del

M: male; F: female; nfvFTLD: non-fluent variant of FTLD; bvFTD: behavioural variant of FTD.

Both, *p62 KD* SH-SY5Y cells and p62 mutant fibroblasts showed a depolarization in response to the F_0_-F_1_-ATP synthase (ATPase or complex V) inhibitor oligomycin (2 µg/ml), suggesting a reverse mode function for the ATPase (Fig. 1E and F). Subsequent inhibition of complex I by rotenone (5 µM) caused a rapid loss of potential in all cells (Fig. 1E and F) and a complete depolarisation was assessed by addition of the mitochondrial uncoupler carbonyl cyanide-4-(trifluoromethoxy) phenylhydrazone (FCCP) (1 μM) (Fig. 1E and F). These data suggest that ΔΨ_m_ in *p62 KD* cells is partially maintained by ATP hydrolysis by the ATPase (Fig. 1E and F).

### Mitochondrial respiration is inhibited in p62 deficient cells and this is associated with limited substrates for the ETC

**Figure 2:**
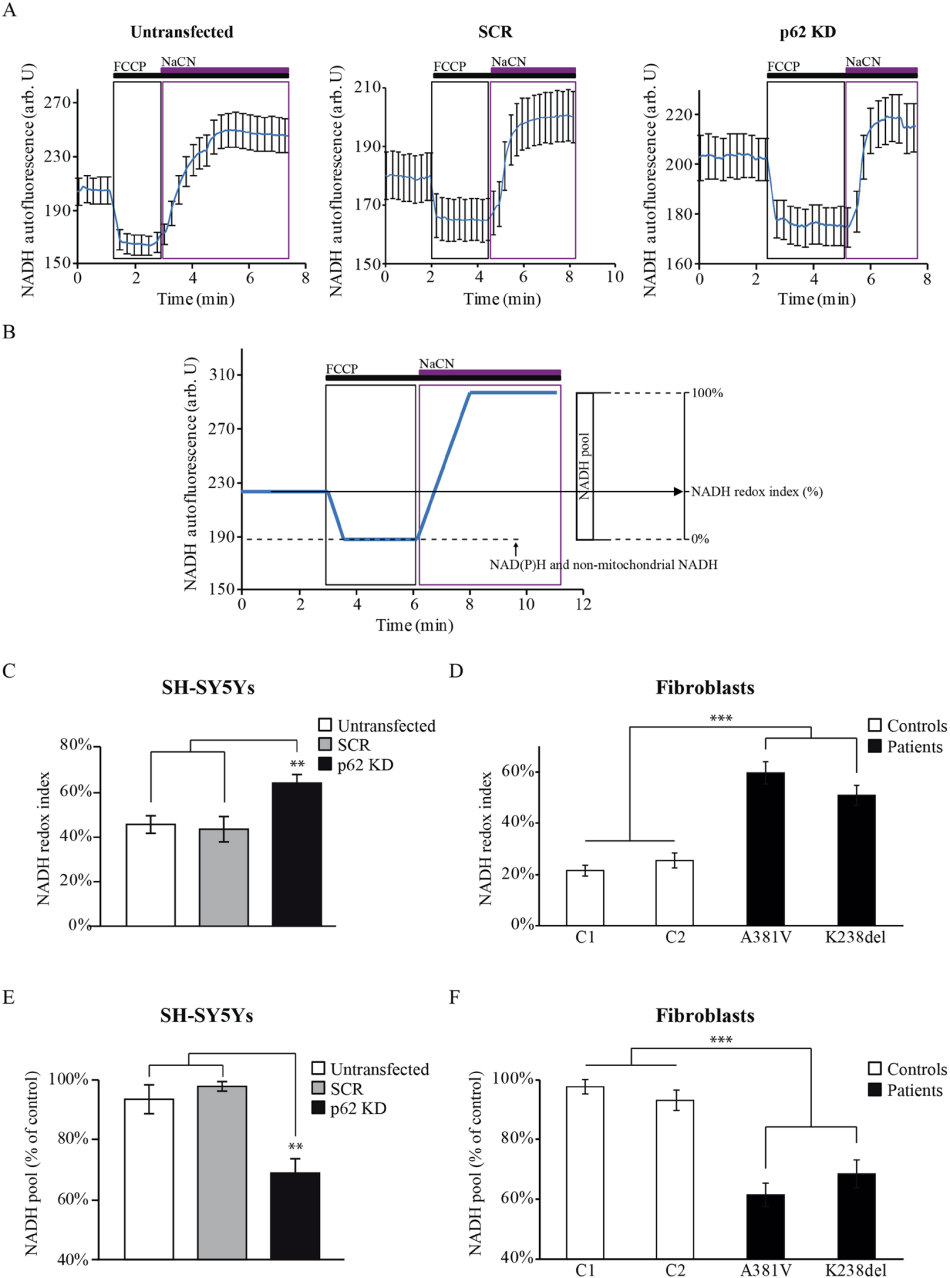
p62 deficiency disrupts the NADH homeostasis. (**A**) Time-course representative traces of NADH autofluorescence from untransfected, SCR and *p62 KD* in SH-SY5Y cells. The uncoupler FCCP (1 μM) maximises mitochondrial respiration and therefore minimises mitochondrial NADH (0%). NaCN (1 mM) was then added to block mitochondrial respiration and thereby maximise mitochondrial NADH (100%). The traces represent the mean of at least 20 cells on a single coverslip ±SEM. (**B**) Graphical description of the NADH homeostasis analysis by monitoring the NADH autofluorescence in cells. NADH redox indexes were obtained by calculating the initial NADH autofluorescence when the minimum NADH autofluorescence is normalised to 0% and the maximum to 100%. Both the NADH redox index (the initial redox level expressed as percentage of the range) and the NADH pool are described graphically. (**C,D**) NADH redox indexes from untransfected, SCR and *p62 KD* SH-SY5Y cells (**C**) and control and p62 deficient fibroblasts (**D**) representing the mean of at least 3 independent experiments ±SEM. In all cases ** indicates p < 0.01 and ***indicates p < 0.001 compared with the values in control cells. (**E,F**) The NADH pool was expressed as absolute values between maximal and minimal respiration in untransfected, SCR and *p62 KD* SH-SY5Y cells (**E**) and control and p62 mutant fibroblasts (**F**). Data represent the mean of at least 3 independent experiments ±SEM. In all cases **indicates p < 0.01 and ***indicates p < 0.001 compared with the values in control cells.

**Figure 3:**
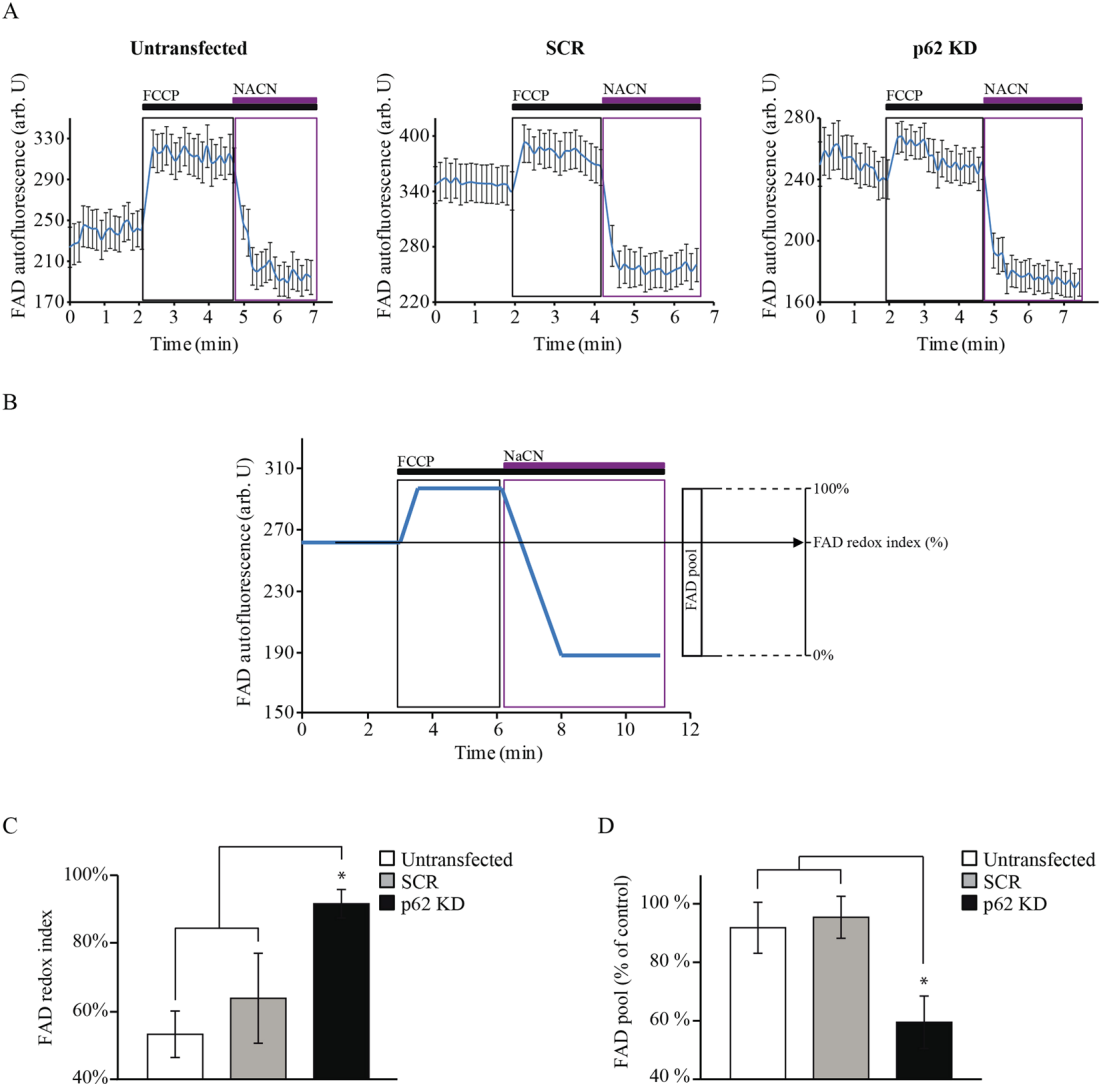
Altered FAD homeostasis is reflected in p62 deficient cells. (**A**) Time-course representative traces of FAD autofluorescence from untransfected, SCR and *p62 KD* SH-SY5Y cells. Addition of FCCP (1 μM) maximised respiration and thereby increased FAD autofluorescence to maximal levels (100%). Addition of NaCN (1 mM) then inhibited respiration and reduced the FAD autofluorescence to a minimum (0%). The traces represent the mean of at least 20 cells on a single coverslip ±SEM. (**B**) Graphical description of the FAD homeostasis analysis by monitoring the FAD autofluorescence using confocal microscopy in p62 deficient cells compared to controls. FAD redox indexes were obtained by calculating the initial FAD autofluorescence when the minimum FAD autofluorescence is normalised to 0% and the maximum to 100%. The FAD redox index generation (the initial redox level expressed as percentage of the range) and the FAD pool are described graphically. (**C**) FAD redox indexes from untransfected, SCR and *p62 KD* SH-SY5Y cells representing the mean of at least 3 independent experiments ±SEM. In all cases * indicates p < 0.05 compared with the values in the corresponding control cells. (**D**) The FAD pool was expressed as absolute values between maximal and minimal respiration in untransfected, SCR and *p62 KD* SH-SY5Y cells. Data represent the mean of at least 3 independent experiments ±SEM. * indicates p < 0.05 compared with the values in the corresponding control cells.

The activity of the mitochondrial electron transport chain (ETC) and the rate of substrate supply can be estimated by measurement of mitochondrial NADH and FAD autofluorescence as previously shown^22,23^ (Figs 2 and 3). Figure 2A shows representative average traces for NADH autofluorescence in untransfected, SCR-transfected and transient *p62 KD* SH-SY5Ys cells (Fig. 2A). Then, the NADH redox index was estimated as represented in Fig. 2B. The obtained NADH redox index for the transient *p62 KD* SH-SY5Y cells was significantly higher (63 ± 4%, n = 5) compared to either untransfected (45 ± 4%, n = 5) or SCR-transfected (43 ± 6%, n = 5) cells (Fig. 2C). Equivalent results were obtained in p62 mutant fibroblasts when compared to healthy controls (NADH redox index in patient 1 = 59 ± 5%, n = 9; patient 2 = 50 ± 4%, n = 6; control 1 = 22 ± 2%, n = 8; control 2 = 26 ± 3%, n = 8) (Fig. 2D). The analysis of the FAD autofluorescence (Fig. 3A) was used to generate the FAD redox index (Fig. 3B and C) which was higher in the *p62 KD* SH-SY5Y cells (91 ± 4%, n = 4) compared to untransfected (53 ± 6%; n = 4) and SCR (63 ± 13%; n = 4) cells (Fig. 3C). Increased NADH and FAD redox indexes in p62 deficient cells reflects inhibition of complex I-driven respiration and suggest more activated complex II dependent respiration as a compensatory mechanism. We were unable to measure the FAD redox state in fibroblasts from both patients and controls due to the very low level of FAD autofluorescence in these cells. Mitochondrial NADH pool obtained as represented in Fig. 2B was found to be reduced in the *p62 KD* cells (68 ± 5%, n = 5) compared to untransfected (94 ± 5%, n = 5) and SCR (97 ± 2%, n = 5) cells (Fig. 2E) and in the p62 mutant fibroblasts when compared to the control fibroblasts (patient 1: 61 ± 4%, n = 9; patient 2: 68 ± 5%, n = 6; control 1: 98 ± 2%, n = 11; control 2: 93 ± 3%, n = 11) (Fig. 2F) indicating a lack of substrates. Along with this observation, lower FAD pool levels were also found in the *p62 KD* cells (59 ± 9%, n = 4) compared to untransfected (91 ± 9%, n = 4) and SCR (95 ± 7%, n = 4) confirming an inhibition in complex I and reduced substrate availability (Fig. 3D). Further analysis of complex I activity using an activity microplate assay confirmed the inhibition of complex I in the p62 mutant fibroblasts compared to controls (patient 1 = 74 ± 1%, n = 3; patient 2 = 55 ± 8%, n = 3; control 1 = 99 ± 7%, n = 3; control 2 = 101 ± 4%, n = 3) (Fig. S3). It is possible that the p62 deficient cells try to compensate the inhibition of complex I-driven respiration through activation of complex II as reflected by the lower FAD pool levels in the p62 deficient cells (Fig. 3D).

### p62 deficient cells exhibit increased cytosolic ROS production

**Figure 4:**
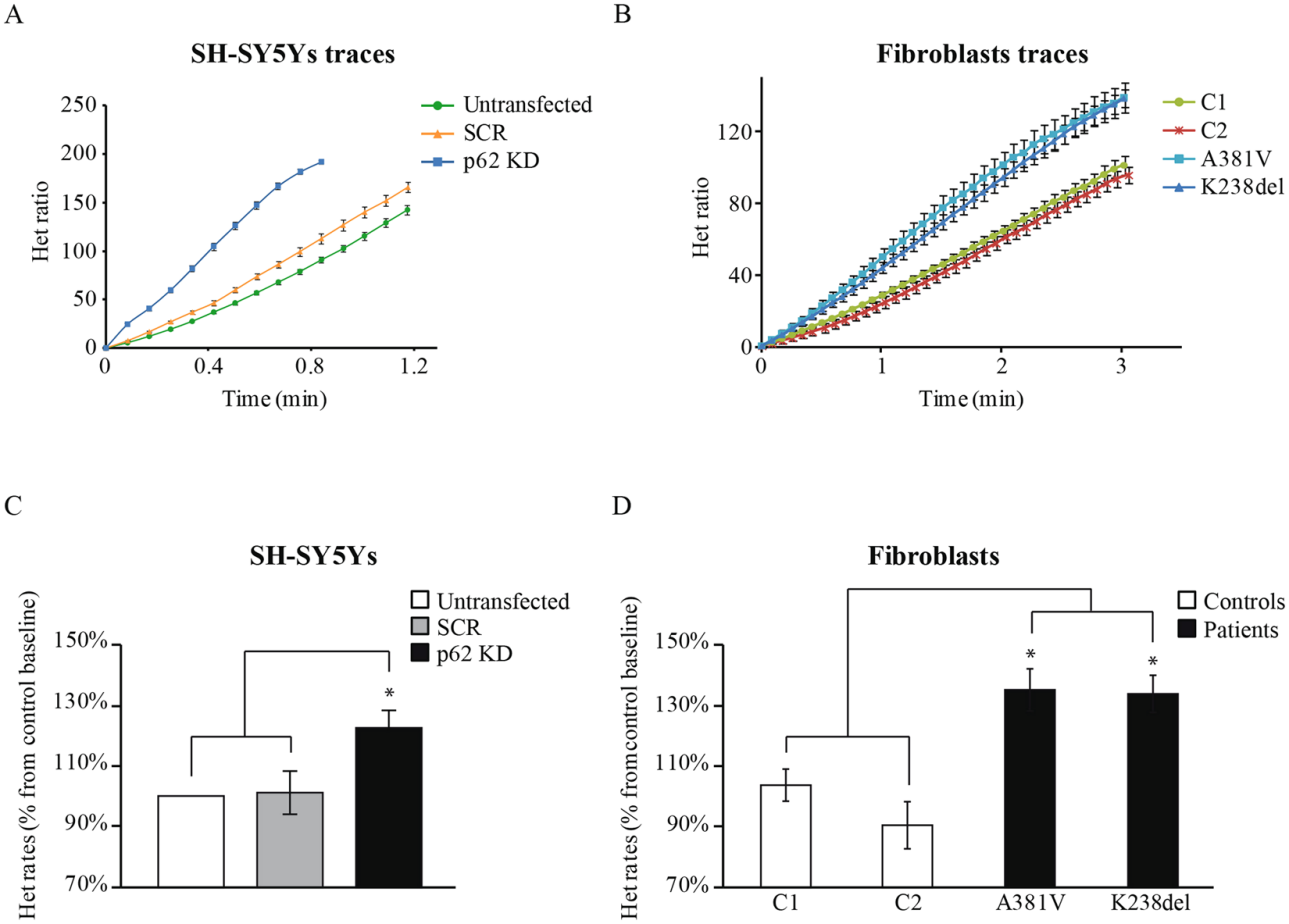
p62 deficiency results in increased oxidative stress. (**A,B**) Cellular oxidative stress was evaluated analysing the oxidation rates of the superoxide indicator dihydroethidium (Het) which initially exhibits cytosolic blue fluorescence and when oxidised, it shows bright red fluorescence in the nucleus. These changes can be detected by live-cell imaging. Time-course representative traces showing the Het oxidation slopes in untransfected, SCR and *p62 KD* SH-SY5Y cells (**A**) and fibroblasts from control donors and patients carrying the *p62* pathogenic mutations mentioned above (**B**). In all cases the traces represent the mean of at least 20 cells on a single coverslip ±SEM. (**C,D**) Het oxidation rates from untransfected, SCR and *p62 KD* SH-SY5Y cells (**C**) and control and p62 mutant fibroblasts **(D)**. Rates were calculated as the change in fluorescence over a period of time and are expressed as percentage of a basic rate. Data represent the mean of at least 3 independent experiments ±SEM. In all cases * indicates p < 0.05 compared with the values in control cells.

Altered mitochondrial function could be linked to an overproduction of cytosolic reactive oxygen species (ROS). Cytosolic ROS production was analyzed in the p62 deficient cells using dihydroethidium (Het). Figure 4 shows representative Het fluorescence rates in SH-SY5Y cells (Fig. 4A) and in fibroblasts (Fig. 4B). The *p62 KD* cells showed higher Het rates (122 ± 6%, n = 5) compared to the untransfected (100 ± 0%, n = 5) or SCR cells (101 ± 7%, n = 5) (Fig. 4C). Equivalent results were obtained in the p62 mutant fibroblasts (patient 1 = 135 ± 7%, n = 4; patient 2 = 133 ± 6%, n = 4) compared to controls (control 1 = 103 ± 5%, n = 4; control 2 = 90 ± 8%, n = 4) (Fig. 4D). Together, all the p62 deficient cells showed cytosolic ROS overproduction in agreement with the inhibited respiration found in these cells.

### Nrf2 activators restored the phenotype in mutant p62 cells

**Figure 5:**
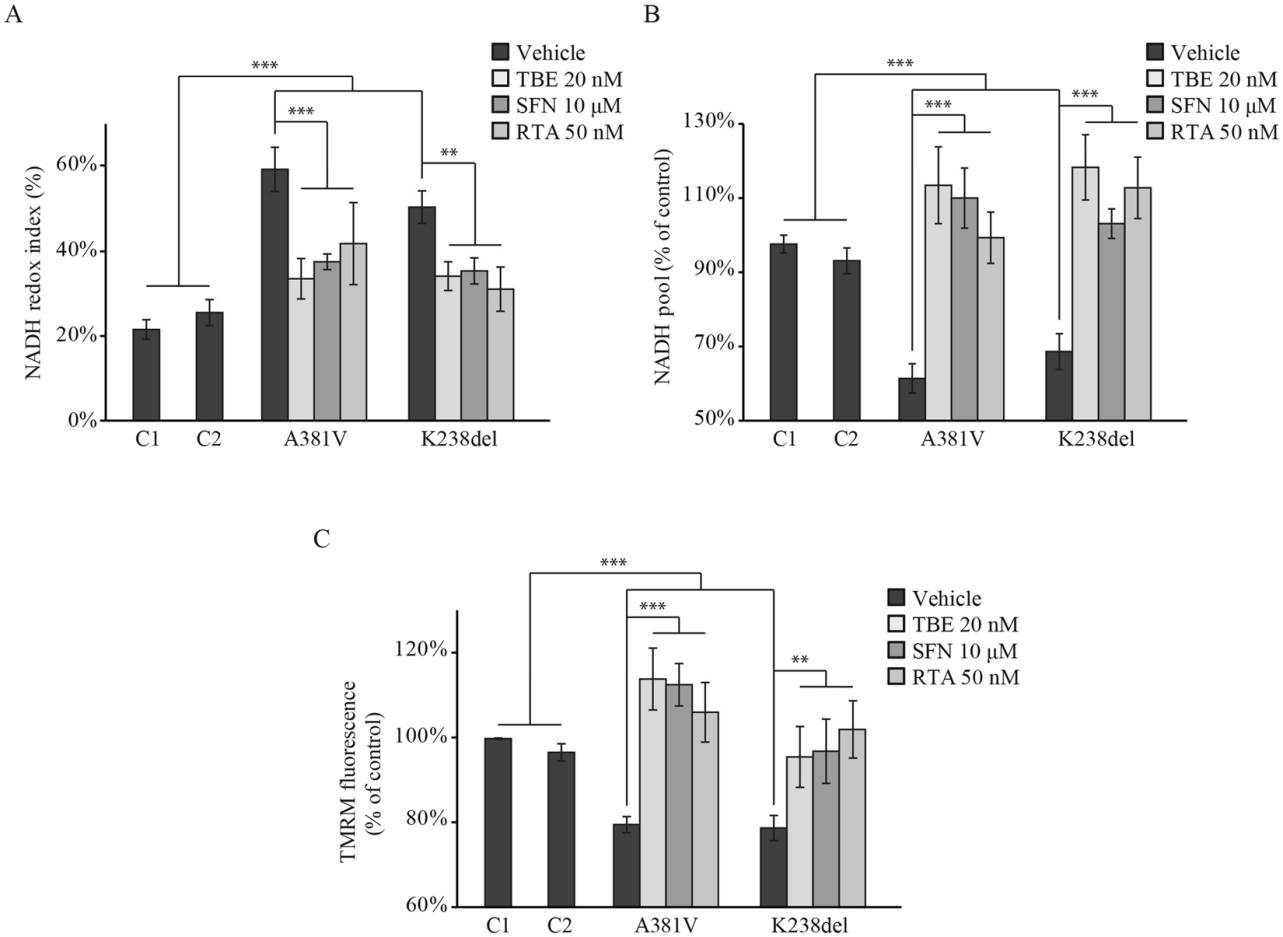
Nrf2 activators restore the p62 deficient phenotype. (**A)** NADH redox index was evaluated after incubation with the Nrf2 activators TBE-31(TBE, 20 nM), sulphoraphane (SFN, 10 μM) and the synthetic triterpenoid RTA-408 (RTA, 50 nM). Cells were plated on 25 mm coverslips in 6 well plates. When they reached 70% confluency, cells were treated 24 hours with TBE, SFN and RTA separately. NADH redox index was calculated as the basal level relative to maximal respiration after FCCP (1 mM) (0%) and inhibited respiration after NaCN (1 mM) (100%) (see Fig. 2B) in fibroblasts from patients carrying the A381V and K238del pathogenic mutations in *p62* compared to two control fibroblasts (C1, C2) in presence of the Nrf2 activators. All data represents the mean of at least 3 independent experiments ±SEM. In all cases ** indicates p < 0.01 and *** indicates p < 0.001 compared with the values in control cells. (**B**) NADH pool expressed as absolute values between maximal and minimal respiration (see Fig. 2B) in control and patient fibroblasts in presence of the Nrf2 activators. Experimental conditions were the same as in (**A**). All data represents the mean of at least 3 independent experiments ±SEM. In all cases *** indicates p < 0.001 compared with the values in control cells. (**C**) The ΔΨ_m_ was analyzed in fibroblasts from patients and controls upon activation of Nrf2. Experimental conditions were the same as in (**A**). All data represents the mean of at least 3 independent experiments ±SEM. In all cases ** indicates p < 0.01 and *** indicates p < 0.001 compared with the values in control cells.

Previous work from our group showed that the nuclear factor erythroid-derived 2 (NF-E2)-related factor 2 (Nrf2) directly regulates cellular energy metabolism by modulating the availability of substrates for mitochondrial respiration^24,25^. It was reported that p62 and Nrf2 form a positive feed-forward regulatory loop, whereby p62 activates Nrf2 by competing for binding with the negative regulator Kelch-like ECH associated protein 1 (Keap1), and Nrf2 activates the transcription of p62^26^. These observations, together with the fact that both models of p62 deficiency show similar mitochondrial alterations to the ones we had previously shown in Nrf2-deficient cells^24^ prompted us to evaluate the role of Nrf2 activation on mitochondrial function in p62-deficient cells. To this end we used three different Nrf2 activators, the synthetic acetylenic tricyclic bis(cyanoenone) (TBE-31), the naturally occurring isothiocyanate sulforaphane (SFN) and the synthetic triterpenoid RTA-408 (RTA). Each of the three pharmacological Nrf2 activators rescued the respiration deficiencies in the fibroblasts carrying the p62 mutations (Fig. 5A and B). The NADH redox index was significantly reduced in p62 mutant fibroblasts treated with Nrf2 activators compared to the same fibroblasts without treatment (patient 1 TBE = 34 ± 5%, n = 6; patient 2 TBE = 34 ± 3%, n = 7; patient 1 SFN = 37 ± 2%, n = 5; patient 2 SFN = 35 ± 3%, n = 5; patient 1 RTA = 42 ± 10%, n = 3; patient 2 RTA = 31 ± 5%, n = 4) (Fig. 5A) and the NADH pool was restored reaching equivalent values to the control fibroblasts (Fig. 5B) (patient 1 TBE = 113 ± 10%, n = 6; patient 2 TBE = 118 ± 9%, n = 7; patient 1 SFN = 110 ± 8%, n = 5; patient 2 SFN = 103 ± 4%, n = 5; patient 1 RTA = 99 ± 7%, n = 3; patient 2 RTA = 112 ± 8%, n = 4). Consequently, the restoration of the mitochondrial NADH pool increased the ΔΨ_m_ in the p62 fibroblasts (Fig. 5C), (patient 1 TBE = 114 ± 7%, n = 7; patient 2 TBE = 95 ± 7%, n = 4; patient 1 SFN = 113 ± 5%, n = 7; patient 2 SFN = 97 ± 8%, n = 5; patient 1 RTA = 106 ± 7%, n = 3; patient 2 RTA = 102 ± 7%, n = 5). Taken together, these results suggest that Nrf2 activation restores cellular metabolism in the p62-deficient cells by increasing the availability of substrates for mitochondrial respiration.

### p62 deficiency is associated with higher pentose phosphate pathway (PPP) activity

**Figure 6:**
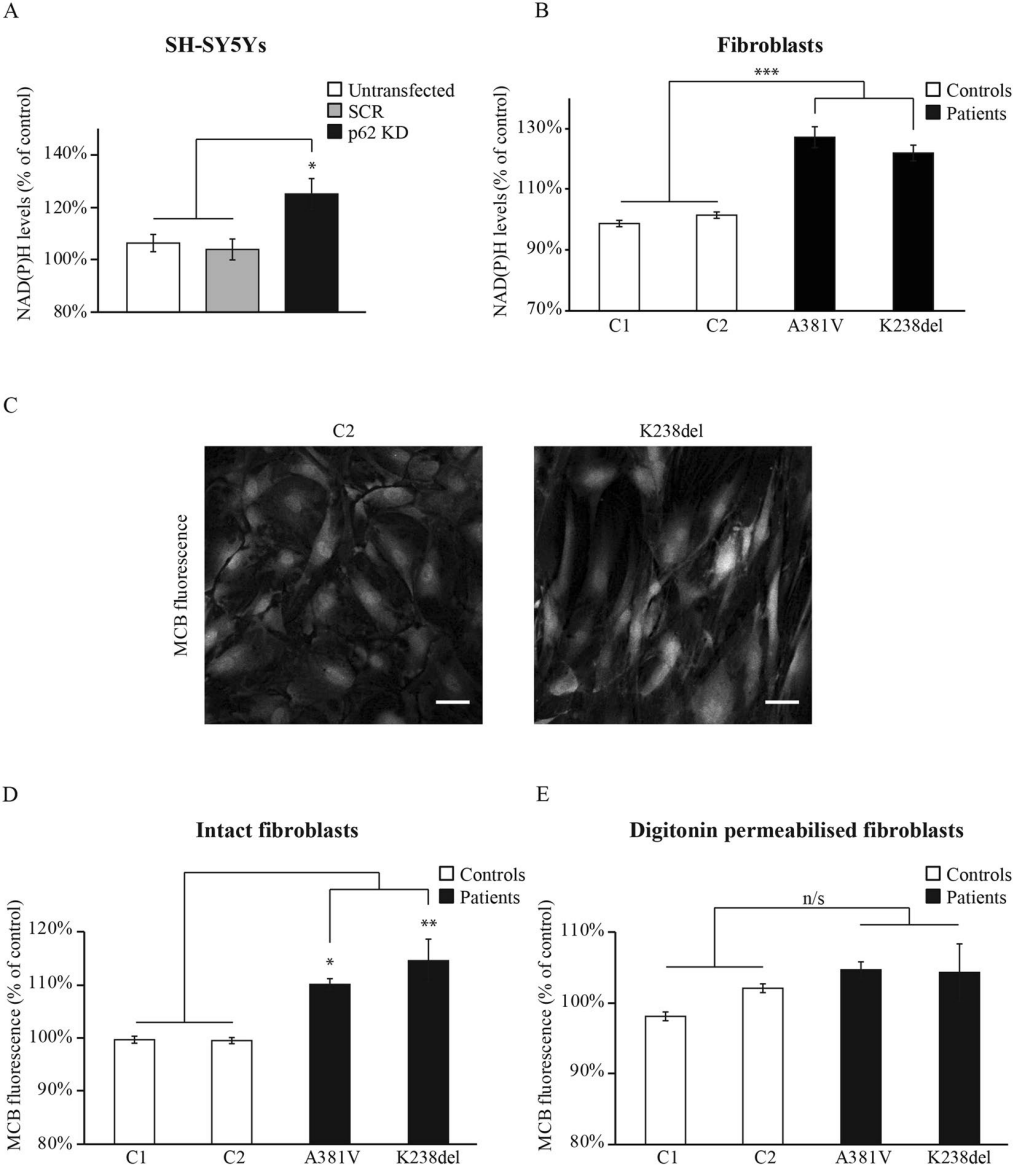
Pentose phosphate pathway activity is increased upon p62 deficiency. (**A,B**) NAD(P)H levels were obtained through the analysis of NADH autofluorescence. The NAD(P)H values were calculated by subtracting the background from the minimum fluorescence after addition of FCCP (1 μM) which oxidises all the mitochondrial NADH minimising the NADH fluorescence (see Fig. 2B). NAD(P)H levels from untransfected, SCR and *p62 KD* SH-SY5Y cells (**A**) and control and p62-deficient fibroblasts (**B**) representing the mean of at least 3 independent experiments ±SEM. * indicates p < 0.05 and *** indicates p < 0.001 compared with the values in control cells. (**C–E**) GSH levels were analyzed by measuring the monochlorobimane (MCB) fluorescence. Representative images showing the cell fluorescence after MCB incubation (50 μM) in fibroblasts from control 2 (C2) and patient 2 carrying the K238del mutation (**C**). Scale bar represents 44 μm. The GSH levels were obtained after evaluation of the MCB fluorescence in fibroblasts from all *p62* mutant carriers and the obtained values were compared to those from the control fibroblasts before (**D**) and after digitonin treatment (40 nM) (**E**). All data represents the mean of at least 3 independent experiments ±SEM. In all cases * indicates p < 0.05 and ** indicates p < 0.01 compared with the values in control cells.

Upon oxidative stress, cells may react by increasing the levels of reduced glutathione (GSH), which is one of the major endogenous antioxidants in the cell. During an oxidative insult, neurons divert part of their glucose pool towards the PPP, thereby increasing the production of NAD(P)H, a necessary cofactor in the regeneration of GSH. PPP activity can be estimated by analysing the basal levels of NAD(P)H autofluorescence after addition of 1 μM FCCP, which maximises respiration (Fig. 2A). This basal fluorescence reflects the non-mitochondrial NADH and the NADPH levels. The NAD(P)H levels in the *p62 KD* SH-SY5Y cells (125 ± 6%, n = 4) were significantly increased compared to untransfected (106 ± 3%, n = 4) and SCR-transfected (104 ± 4%, n = 4) SH-SY5Y cells indicating an increased activity in the PPP due to p62 deficiency (Fig. 6A). Similar results were obtained in the p62 mutant fibroblasts when compared to controls (patient 1 = 127 ± 3%, n > 10; patient 2 = 122 ± 3%, n > 10; control 1 = 99 ± 1%, n > 10; control 2 = 102 ± 1%, n > 10) (Fig. 6B). We found no changes between control and patient fibroblasts in the glucose 6 phosphate dehydrogenase protein levels, a rate limiting enzyme in the PPP, indicating the PPP increased activity observed was not related to higher expression levels of this enzyme (Fig. S2). Finally, using the monochlorobimane (MCB) fluorescent probe to determine GSH levels (Fig. 6C) we found increased cytosolic (Fig. 6D) but not mitochondrial (Fig. 6E) GSH levels in the fibroblasts carrying the p62 mutations (cytosolic: control 1 = 99 ± 1%, n = 7; control 2 = 99 ± 1%, n = 9; patient 1 = 110 ± 2%, n = 5; patient 2 = 114 ± 7%, n = 5; mitochondrial: control 1 = 98 ± 1%, n = 7; control 2 = 102 ± 1%, n = 9; patient 1 = 105 ± 2%, n = 5; patient 2 = 104 ± 11%, n = 5). The increased GSH levels in patients fibroblasts were confirmed using a quantitative fluorometric assay (control 1 = 102 ± 2%, n = 3; control 2 = 98 ± 2%, n = 3; patient 1 = 153 ± 14%, n = 3; patient 2 = 156 ± 21%, n = 3) (Fig. S2). These data, along with the higher NADPH levels, revealed an increased PPP activity in the p62-deficient cells compared to the healthy controls, possibly due to a switch from glycolysis to PPP.

## Discusion

Mitochondria are the main source of energy in neurons due to their limited glycolytic capacity. Harnessing the proton gradient generated in the respiration through complexes I to IV, neurons rely on the oxidative phosphorylation process to generate ATP through the ATP synthase in the mitochondria. Mutations in FTD and ALS have been found to induce impairments in crucial mitochondrial metabolic processes causing a fatal fate for cells, organs and patients^27^. Previous works presented p62 protein as one of the main regulators of mitochondrial function^14,16,17^. In this study, the mitochondrial bioenergetics in p62 deficient cells and fibroblasts from FTD patients carrying independent *p62* mutations was investigated. Reduced p62 levels are associated with reduced ΔΨ_m_ in both p62 SH-SY5Y deficient cells and mutant fibroblasts. These cells also exhibit inhibited mitochondrial respiration through complex I due to the lack of substrates for the ETC complexes and higher superoxide production. The boost in the cytosolic ROS levels leads to activation of the pentose phosphate pathway, thereby increasing the GSH levels in order to counteract the oxidative stress environment.

Mitochondrial dysfunction in ALS has been thoroughly demonstrated^28–30^ but not much is known about this topic in FTD. Recent studies found mitochondrial alterations in FTD cases together with ALS supporting the idea of the FTD/ALS continuum^18–20,31–34^. Other works showing mitochondrial alterations were carried out in different cohorts of patients with variations in the *CHCH10* gene, a common cause of both FTD and ALS^31,35^. A number of studies have demonstrated that mitochondrial activity is compromised in FTD and ALS using animal models and cells from patients^22,36^. In this study, a reduction in the ΔΨ_m_ is observed in the p62 deficient cell models. The ΔΨ_m_ reflects mitochondrial health and function and mitochondrial viability and it is defined as the proton gradient generated in the intermembrane space during respiration. This proton gradient is harnessed by the ATPase to synthesise ATP through the oxidative phosphorylation (OXPHOS) process. Here it is also shown that ATP hydrolysis by the ATPase maintains the ΔΨ_m_ in the p62 deficient cells since these cells exhibited depolarised mitochondria when oligomycin, the inhibitor of the F_1_-F_0_ ATPase, was added. Under normal conditions the process of respiration maintains the ΔΨ_m_ but mitochondria can also hydrolyze the ATP forcing the ATPase to work in a reverse mode in order to maintain the ΔΨ_m_^37^. This has been thoroughly described in other neurodegenerative models in which an inhibition of respiration hampers the normal ΔΨ_m_ maintenance forcing the reversal ATPase^38–40^.

In combination with the reduced ΔΨ_m_ an inhibition in the respiration process was detected in p62 deficient cells. This was reflected by both the elevated NADH redox index and the decreased FAD redox index, pointing to the inhibition of complex I. This inhibition is a consequence of lack of substrates as demonstrated by the lower NADH pool observed in the p62 deficient cells as well as the lower FAD pool. The inhibition of the respiration through the complex I induces an increase in ROS production in the cells. Damaged mitochondrial respiration including reduced complex I and II activities were previously found in mitochondria from p62 KO mice brains^16^. These authors also found an exacerbated oxidative stress due to inhibition of respiration. On the other hand, in 2012 Kwon and colleagues showed mitochondrial impairments in a *p62* KO model reflected in reduced mitochondrial membrane potential and reduced oxygen consumption together with increased cellular oxidant levels^17^. Increased ROS generation has been found to be related to inhibited mitochondrial respiration in neurodegenerative disorders^41–43^. In AD, it has been found that an inhibition in mitochondrial respiration through complex I increased ROS generation^44^. Increased ROS production is also a demonstrated feature in some forms of PD. This has been related to inhibition in the complex I dependent respiration both in cells and PD animal models^39,45,46^.

The results presented herein show that Nrf2 activators restore the inhibition of the respiration and the NADH pool in the p62 deficient cells. Nrf2 is a transcription factor implicated in the activation of antioxidant genes, providing cytoprotection against oxidative stress and inflammation^47^. More recently, new roles regarding the maintenance of mitochondrial function and bioenergetics have been attributed to Nrf2^24,47–49^. It has been shown that cells from Nrf2-deficient mice have impaired mitochondrial function. Specifically, it was demonstrated that Nrf2 is a main regulator in the substrates supply to both complex I and complex II as the rate of NADH and FADH_2_ production was much lower in the Nrf2 KO cells compared to controls^24^. Nrf2 and p62 were shown to intersect through the direct interaction between p62 and Keap1^26^. Under normal conditions Nrf2 is ubiquitinated and degraded by the proteasome as it is bound to the E3-ubiquitin ligase adaptor protein Keap1. When Keap1 is challenged with ROS or electrophiles, Nrf2 degradation is blocked, activating the antioxidant response elements (ARE)-mediated transcription. In the noncanonical pathway of Nrf2 regulation, Keap1 is sequestered by p62 and can no longer bind Nrf2, leading to increased Nrf2 signalling with the subsequent activation of the ARE-mediated transcription. Our findings point to a disruption in the Keap1 sequestration by p62, leading to enhanced degradation of Nrf2. The consequence would be inhibition in respiration due to low Nrf2 levels, which could be restored by Nrf2 pharmacological activators.

Finally, it is shown that p62 deficiency is associated to an increased activity in the pentose phosphate pathway as demonstrated by the elevated levels of NAD(P)H and reduced glutathione. It has been demonstrated that elevated ROS levels in cells induce the activation of the PPP to generate the antioxidant GSH^50^. The expression levels of the rate limiting enzyme responsible for GSH biosynthesis, the glutamate-cysteine ligase, are very low in neurons. This fact makes neurons more vulnerable to oxidative stress as the GSH-dependent antioxidant system is very weak in these cells. Neurons can compensate their oxidative stress vulnerability by diverting part of the glucose pool to the PPP, which generates NAD(P)H and increases the GSH levels^51^. It is possible that p62 deficient cells divert part of their glucose to the PPP to increase GSH levels after an oxidative stress stimulus, as has been shown in neurons^52^.

In summary, our results show that p62 deficiency induces mitochondrial respiration inhibition by deficient delivery of substrates to the mitochondrial complexes, which subsequently results in elevated cytosolic oxidative stress. The rise in the oxidative stress could partially trigger glucose diversion to the PPP in order to increase the GSH levels.

Even though our results were obtained using fibroblasts from FTD patients, they have been also validated using the SH-SY5Y *p62 KD* model, suggesting a loss-of-function effect upon p62 deficiency. This point makes the results applicable not only to FTD patients carrying p62 mutations but also to ALS patients with mutations in p62, and therefore supports the idea of FTD/ALS continuum. The data presented here along with recent works showing functional mitochondrial impairments linked to mutations causing both FTD and ALS provides significant basis to support the hypothesis that mitochondrial dysfunction is involved in the underlying pathogenic mechanisms within the FTD/ALS spectrum. Additionally, the pharmacological activation of Nrf2 needs to be further explored as therapeutic tool for the p62-associated ALS/FTD prevention and treatment.

## Methods

All methods were performed following the relevant guidelines and regulations approved by the local ethical review committee from the National Hospital for Neurology and Neurosurgery and the Institute of Neurology.

### Donors

Written informed consent was obtained from the donors for publication of their individual details. The consent form is held by the authors’ institution within the patients’ clinical notes and is available for review by the Editor-in-Chief. Donors gave written consent and the project was approved by the local ethical review committee from the National Hospital for Neurology and Neurosurgery and the Institute of Neurology (London, UK). Fibroblasts from two patients carrying independent *p62* mutations and two non-related healthy donors used as controls were generated from a 4-mm skin punch biopsy taken under local anesthetic following informed consent. Biopsies were dissected into ~1-mm pieces and cultured in 5-cm^2^ petri dishes in DMEM, 10% FBS, 1% L-Glutamine until fibroblasts were seen to grow out from the explants. When fibroblasts reached confluency, they were detached from culture dishes using TrypleE from Thermo Fisher Scientific (Waltham, MA, USA) and transferred to larger culture vessels for further expansion and cryopreservation. Age, sex, age of onset, clinical features, clinical diagnosis, and *p62* mutations carried by the patients as well as healthy donors characteristics are given in Table 1. Family trees from patient 1 and patient 2 are provided in (Supplementary Fig. S1).

### Cell culture

Human neuroblastoma (SH-SY5Y) cells were purchased from the European Collection of Cell Cultures (Health Protection Agency, Salisbury, UK) and maintained as previously described^53^. Unless otherwise stated, SH-SY5Y cells and fibroblasts were seeded at a density of 4 × 10^4^ cells/cm^2^, grown to 75–80% confluence and maintained at 37 °C and 5% CO2 in Dulbecco’s modified Eagle’s medium (DMEM) medium supplemented with 10% (v/v) foetal bovine serum (FBS), 2 mM L-glutamine and 1% (v/v) penicillin/streptomycin.

### Immunoblotting and antibodies

Cells lysates were obtained after 2 minutes of incubation with the lysis buffer NP-40 (50 mM Tris-base pH 7.4, 150 mM NaCl, 0.5% Nonidet P-40, 1 mM EDTA, protease inhibitors cocktail) and the lysates were immediately frozen. 10 μg of denatured protein previously estimated (Pierce BCA Protein Assay Kit, Thermo Fisher, Waltham, MA, USA) from each sample were loaded in a precast 4–20% Tris-Glycine SDS-PAGE (Bio Rad Laboratories, Inc., Hercules, CA). After electrophoresis, proteins were transferred to 0.45 μm PVDF membranes (IPVH00010, Immobilon Millipore) and identified by the appropriate primary and secondary antibodies and visualised using Enhanced Chemiluminescence (ECL Clarity; BioRad) with the ImageQuant LAS4000 (GE Healthcare). Anti-human p62 rabbit monoclonal antibody (1:20000) and anti-human glucose 6 phosphate dehydrogenase (1:1000) were obtained from Abcam (Cambridge, UK). Anti-human β-Actin rabbit polyclonal antibody (1:5000) was obtained from Sigma-Aldrich (Poole, UK).The anti-rabbit secondary antibodies (1:5000) coupled to horseradish peroxidase and bovine immunoglobulins (IgG) were from Bio-Rad (Richmond, CA).

### Plasmids and reagents

The non-targeting scramble siRNA, the targeted siRNA (siGenome SMARTpool) against human p62 and the Dharmafect transfection reagent were purchased from Dharmacon, Thermo Fisher Scientific (Waltham, MA, USA). The siRNA transfection was performed following the manufacturer’s instructions once the plated cells reached 60% confluence. Cells were ready for subsequent experiments after 48 h post transfection 37 °C and 5% CO_2_.

### Nrf2 activators

Three different activators for the transcription factor Nrf2 were used: the synthetic acetylenic tricyclic bis(cyanoenone) TBE-31^54^, the triterpenoid RTA-408 (RTA)^55^, and the naturally occurring isothiocyanate sulforaphane (SFN)^56^. The concentration of each compound was optimized based on the potency in inducing NAD(P)H:quinone oxidoreductase 1 (NQO1), a prototypic Nrf2 target gene, without causing any cytotoxicity, using a quantitative bioassay^57^. The compounds were prepared as stock solutions in acetonitrile and diluted (1:1000) in the cell culture medium, such that the final concentration of acetonitrile was maintained at 0.1% (v/v). Cells were exposed to the Nrf2 activators for 24 h.

### Measurement of mitochondrial membrane potential (ΔΨ_m_)

ΔΨ_m_ was measured as was described previously^22^. Briefly, cells were plated on 25 mm coverslips and loaded with 40 nM tetramethyl-rhodamine methyl ester (TMRM) in a HEPES-buffered salt solution (HBSS) (composed of 156 mM NaCl, 3 mM KCl, 2 mM MgSO_4_, 1.25 mM KH_2_PO_4_, 2 mM CaCl_2_, 10 mM glucose and 10 mM HEPES; pH adjusted to 7.35 with NaOH) for 40 minutes at room temperature and keeping the dye present in the chamber at the time of recording. TMRM is a cell-permeant fluorescent dye used in the redistribution mode to assess ΔΨ_m_, and therefore a reduction in TMRM fluorescence represents ΔΨ_m_ depolarization. Confocal images were obtained using a Zeiss 710 VIS CLSM (Zeiss, Oberkochen, Germany) equipped with a META detection system and a × 40 oil immersion objective. TMRM was excited using the 560 nm laser line and fluorescence was measured above 580 nm. The Z-stack images were analyzed using the Volocity software (PerkinElmer, Waltham, MA) and TMRM values for control cases were set to 100% and the p62 deficient cells values were expressed relative to controls. For analysis of response to mitochondrial toxins, images were recorded in a time course-dependent manner from a single focal plane and analyzed using ZEN Zeiss software (Zeiss).

### Measurement of NADH-FAD autofluorescence

NADH autofluorescence was measured using an epifluorescence inverted microscope equipped with a 20× fluorite objective. Excitation light at a wavelength of 350 nm was provided by a Xenon arc lamp, with the beam passing through a monochromator (Cairn Research, Faversham, Kent, UK). Emitted fluorescence light was reflected through a 455 nm long-pass filter to a cooled CCD camera (Retiga, QImaging, Surrey, BC, Canada) and digitised to 12 bit resolution. Imaging data were collected and analyzed using software from Andor (Belfast, UK). FAD autofluorescence was monitored using a Zeiss 710 VIS CLSM equipped with a META detection system and a × 40 oil immersion objective. Excitation was measured using the 454 nm Argon laser line and fluorescence was measured from 505 to 550 nm. Illumination intensity was kept to a minimum (at 0.1–0.2% of laser output) to avoid phototoxicity and the pinhole set to give an optical slice of ~2 μm.

### Complex I activity assay

The NADH dehydrogenase activity of isolated complex I was measured using the complex I enzyme activity microplate assay kit (Abcam, ab109721). Cell lysis was carried out using the lysis buffer provided by the manufacturer and left on ice for 20 min to allow protein extraction. Samples were then centrifuged at 12, 000 × g for 20 min at 4 °C and total protein concentration in the supernatant was estimated using the Pierce BCA Protein Assay Kit (Thermo Fisher, Waltham, MA, USA). Samples were then diluted in Incubation Solution (provided by the manufacturer) to reach a final protein concentration of 0.5 μg/μl. 200 μl of each sample were transferred to each well of the microplate containing immobilised anti-complex I antibody bound to the wells and incubated for 3 hr at RT. After the incubation period, the wells were rinsed twice in 300 μl of Buffer, provided by the manufacturer, and 200 μl of Assay Solution containing NADH and a reporter dye were added to each well. NADH dehydrogenase activity was determined by measuring the oxidation of exogenous NADH to NAD^+^, coupled to the 1:1 reduction of the reporter dye of which product concentration was proportional to the increase in absorbance at 450 nm, measured over time using a spectrophotometer. Complex I activity was expressed as the rate of increase in absorbance per amount of sample loaded in the well.

### ROS measurements

Cellular ROS generation was measured using dihydroethidium (Het, DHE; 2 μM for superoxide) from Life Technologies (Paisley, UK). All imaging was performed in HBSS and the dye was present in the solution during the experiment. No preincubation (“loading”) was used for Het to limit the intracellular accumulation of oxidized products. Fluorescence images were collected with a 12-bit resolution cooled CCD camera coupled to an epifluorescence inverted microscope equipped with a 20× fluorite objective (Leica Microsystems). The excitation wavelength for the oxidised form (ethidium) was 530 nm collected at 605 nm while excitation to measure changes in the reduced form (hydroethidium) was 380 nm collecting the emission at 405–470 nm. Ratiometric Het fluorescence was recorded with excitation light at 380 and 530 nm. All imaging data were collected and analyzed using the Metamorph software (Molecular Devices, US).

### GSH measurements

The reduced glutathione (GSH) levels were determined in live cells after incubation with 50 μM monochlorobimane (MCB) for 40 minutes at room temperature in HBSS until a steady state was reached. MCB is a nonfluorescent probe until conjugated with GSH. Once the cytosolic GSH was analyzed, HBSS was removed and a hypotonic medium (135 mM KCl, 10 mM NaCl, 20 mM HEPES, 0.5 mM KH_2_PO_4_, 1 mM MgCl_2_, 5 mM EGTA and 1.86 mM CaCl_2_ at pH 7.1) including digitonin (40μM) was added to permeabilise the cells allowing us to measure the mitochondrial GSH. The fluorescence images of the MCB-GSH adduct were acquired using the cooled CCD imaging system as described above using excitation at 380 nm and emission at >400 nm. The fluorescence was then quantified using the Metamorph analysis software (Molecular Devices, US). Additionally, quantitative GSH analysis was performed in live cells in a modified protocol as was previously described^58^. Briefly, cells were plated in 96 well plates. Once they reached 70–80% of confluence they were loaded for 30 minutes with 2 mM MCB allowing the formation of the MCB-GSH fluorescent adduct inside cells. The fluorescence was then quantified using the Varioskan plate reader (Thermo Fisher, Waltham, MA, USA) with excitation wavelength at 380 nm and emission at 470 nm. Fluorescence values were then related to the protein content (Pierce BCA Protein Assay Kit, Thermo Fisher, Waltham, MA, USA).

### Statistical Analysis

Data were generated from a minimum of three independent replicate per experiment (n ≥ 3) performed in different days. Each replicate consisted of at least 1 coverslip per condition where a number of 15–30 cells per coverslip were analyzed. Statistical analysis and exponential curve fitting were performed using GraphPad Prism 6.01 (GraphPad Software, La Jolla, CA) software. Statistical significance for multiple comparisons was performed by one-way ANOVA followed by Fisher’s LSD correction. All results are related to healthy control fibroblasts or untransfected cells accordingly and expressed as percentage. In all cases, *P* < 0.05 was considered significant (*p < 0.05, **p < 0.01, ***p < 0.001). For all graphs, error bars represent mean ± SEM.

### Ethical Approval and Informed Consent

Primary fibroblast lines were generated from skin punch biopsies. Donors gave written consent, and the project was approved by the local ethical review committee from the National Hospital for Neurology and Neurosurgery and the Institute of Neurology.

## Electronic supplementary material

Supplementary information
