# Author Correction: Pathogenic *p62/SQSTM1* mutations impair energy metabolism through limitation of mitochondrial substrates

**DOI:** 10.1038/s41598-018-22152-9

**Published:** 2018-03-01

**Authors:** Fernando Bartolome, Noemi Esteras, Angeles Martin-Requero, Claire Boutoleau-Bretonniere, Martine Vercelletto, Audrey Gabelle, Isabelle Le Ber, Tadashi Honda, Albena T. Dinkova-Kostova, John Hardy, Eva Carro, Andrey Y. Abramov

**Affiliations:** 1 Neurodegenerative Disorders group, Instituto de Investigacion Hospital 12 de Octubre (i+12), Av Cordoba, Madrid, 28041 Spain; 2 Biomedical Research Networking Centre on Neurodegenerative Diseases (CIBERNED), Madrid, Spain; 3 Department of Molecular Neuroscience, UCL Institute of Neurology Queen Square, London, WC1N 3BG UK; 4 Department of Cellular and Molecular Medicine, Centro de Investigaciones Biológicas (CSIC), Ramiro de Maeztu 9, Madrid, 28040 Spain; 5 Biomedical Research Networking Centre on Rare Diseases (CIBERER), Madrid, Spain; 6 Laboratoire d’études des mécanismes cognitifs, EA 3082, Université Lyon 2, Bron, F-69500 France; 7 CHU Nantes, Centre de Mémoire et de Ressource et Recherche (CM2R), Nantes, France; 8 Inserm, CIC 04 Nantes, France; 9 Memory Research and Resources Center, Department of Neurology, Montpellier University Hospital, Montpellier, France; 10 CNR-MAJ, AP-HP, Hôpital de la Pitié-Salpêtrière, Paris, France; 11 ICM, Inserm U1127, CNRS UMR 7225, Sorbonne Universités, UPMC-P6 UMR S 1127 - Hôpital Pitié-Salpêtrière, Paris, France; 12 Department of Chemistry and Institute of Chemical Biology & Drug Discovery Stony Brook University Stony Brook, New York, 11794 USA; 13 Division of Cancer Research, School of Medicine University of Dundee, Dundee, DD1 9SY UK; 14 Reta Lilla Weston Laboratories, London, WC1N 3BG UK

Correction to: *Scientific Reports* 10.1038/s41598-017-01678-4, published online 10 May 2017

The Acknowledgements section in this Article is incomplete.

“We are grateful to the patients and donors without which these work would not have been possible. We thank Patricio Aller and Elena de Blas in the assessment of GSH levels. We also thank the i+12 Proteomic Unit, in particular Ines Garcia-Consuegra for her advice in the Complex I activity measurement in fibroblasts. We also thank Maria Teresa Seisdedos from the CIB Microscopy Unit helping to setup the fluorescence measurements in living cells. FB is supported by a *Sara Borrell* fellowship from the Spanish *Instituto de Salud Carlos III*. N.E. holds a postdoctoral fellowship from the Spanish *Fundación Alfonso Martín Escudero*. This work was supported by a career development award from the MRC (G0700183), by an ALS Association Initiated award (ID#2109) by the Motor Neuron Disease Association, partially by the Wellcome Trust/MRC Joint Call in Neurodegeneration award (WT089698) to the UK Parkinson’s Disease Consortium (UKPDC) whose members are from the UCL/Institute of Neurology, the University of Sheffield and the MRC Protein Phosphorylation Unit at the University of Dundee and supported by the National Institute for Health Research University College London Hospitals Biomedical Research Centre. AMR is supported by grant CTQ2015-66313-R from MINECO, Spain. ILB has received funding from the program “Investissements d’avenir” ANR-10-IAIHU-06, FP7-Neuromics, PHRC FTLD-exomes. EC is supported by grants from the *Instituto de Salud Carlos III* (FIS2012/00486; BA14/00058), FEDER, *Fundación Investigación Médica Mutua Madrileña* (2010/0004), *Fundación Ramón Areces (CIVP16A1825)*, and *CIBERNED*.”

should read:

“We are grateful to the patients and donors without which these work would not have been possible. We thank Patricio Aller and Elena de Blas in the assessment of GSH levels. We also thank the i+12 Proteomic Unit, in particular Ines Garcia-Consuegra for her advice in the Complex I activity measurement in fibroblasts. We also thank Maria Teresa Seisdedos from the CIB Microscopy Unit helping to setup the fluorescence measurements in living cells. FB is supported by a *Sara Borrell* fellowship from the Spanish *Instituto de Salud Carlos III*. N.E. holds a postdoctoral fellowship from the Spanish *Fundación Alfonso Martín Escudero*. This work was supported by a career development award from the MRC (G0700183), by an ALS Association Initiated award (ID#2109) by the Motor Neuron Disease Association, partially by the Wellcome Trust/MRC Joint Call in Neurodegeneration award (WT089698) to the UK Parkinson’s Disease Consortium (UKPDC) whose members are from the UCL/Institute of Neurology, the University of Sheffield and the MRC Protein Phosphorylation Unit at the University of Dundee and supported by the National Institute for Health Research University College London Hospitals Biomedical Research Centre. AMR is supported by grant CTQ2015-66313-R from MINECO, Spain. ILB has received funding from the program “Investissements d’avenir” ANR-10-IAIHU-06, FP7-Neuromics, PHRC FTLD-exomes. EC is supported by grants from the *Instituto de Salud Carlos III* (FIS2012/00486; BA14/00058), FEDER, *Fundación Investigación Médica Mutua Madrileña* (2010/0004), *Fundación Ramón Areces (CIVP16A1825)*, and *CIBERNED*. Thanks to Reata Pharmaceuticals for their support and for providing us the RTA-408 activator.”

