# Supplementary material for: Pathogenic p62/SQSTM1 mutations impair energy metabolism through limitation of mitochondrial substrates
Source: Sci Rep. 2017 May 10;7:1666. doi: 10.1038/s41598-017-01678-4 (PMC5431917; doi:10.1038/s41598-017-01678-4)
Supplement: Supplementary file 1 — Supplementary information [file 41598_2017_1678_MOESM1_ESM.pdf]

Supplementary Information

Pathogenic *p62/SQSTM1* mutations impair energy metabolism through limitation of mitochondrial substrates

Fernando Bartolome, Noemi Esteras, Angeles Martin-Requero, Claire Boutoleau-Bretonniere, Martine Vercelletto, Audrey Gabelle, Isabelle Le Ber, Tadashi Honda, Albena T. Dinkova-Kostova, John Hardy, Eva Carro, Andrey Y. Abramov

Supplementary Figure 1 – Family trees from *p62* mutations carriers.

A – Family tree from patient 1 carrying the p.A381V mutation.

B – Family tree from patient 2 carrying the p.K238del mutation.

In both, affected and non-affected members of the families are labelled in black and white, respectively

Supplementary Figure 2 – Glucose 6 phosphate dehydrogenase levels (G6PD) and GSH measurement related to the protein content in *p62* mutations carriers compared to healthy controls.

A – Immunoblotting showing glucose 6 phosphate dehydrogenase (G6PD) protein levels corresponding to whole cell lysates from fibroblasts from patients carrying *p62* mutations and aged-matched controls C1 and C2 (B). β-actin was used as loading control.

B – Histogram representing the GSH levels in fibroblasts from carriers of the *p62* mutations and healthy controls related to the protein content. The histogram represents the percentage of fluorescence values from patient fibroblasts compared to controls. All data represents the mean of 4 independent experiments carried out in triplicates ± SEM. In all cases * indicates $P<0.05$ compared with the values in control cells.

Supplementary Figure 3 – p62 deficient fibroblasts show inhibited complex I activity compared to healthy controls.

A – Histogram representing the average complex I activity in fibroblasts from carriers of the *p62* mutations and healthy controls. Complex I activity was measured by the rate of increase in absorbance $OD_{450,}$ normalised to control cells from three independent experiments. Data are represented as mean ± SEM. In all cases * indicates $P<0.05$ and *** indicates $P<0.001$ compared with the values in control cells.

A

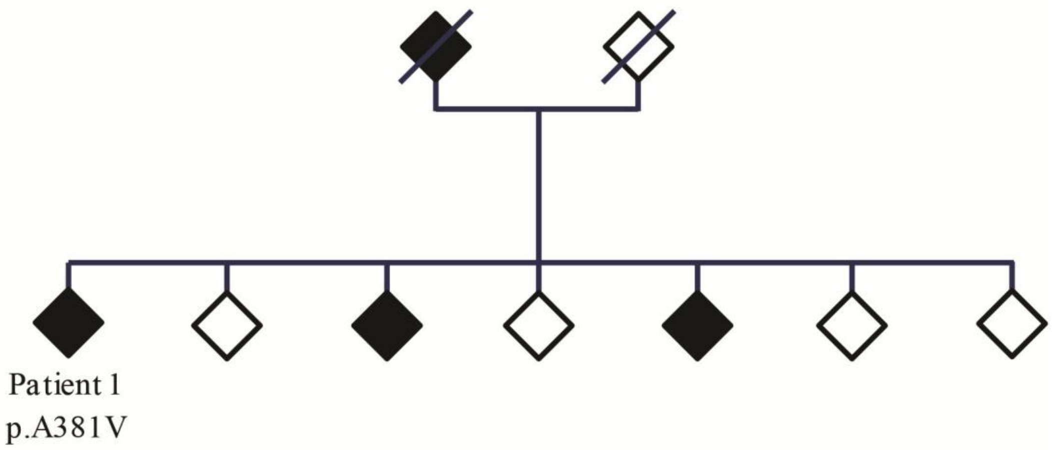

B

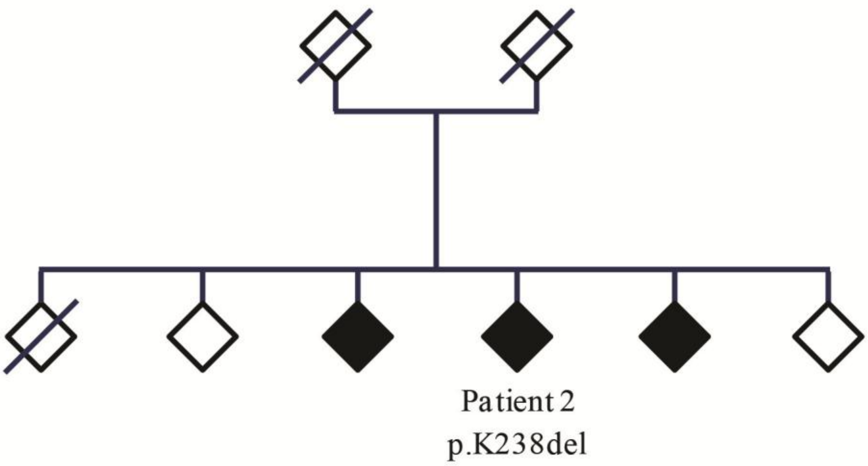

Supplemental Figure 1. Family trees from p62/SQSTM1 mutation carriers. Family tree from patient 1 carrying the p.A381V mutation (A). Family tree from patient 2 carrying the p.K238del mutation (B). In both, affected and non-affected members from the families are labeled in black and white, respectively.

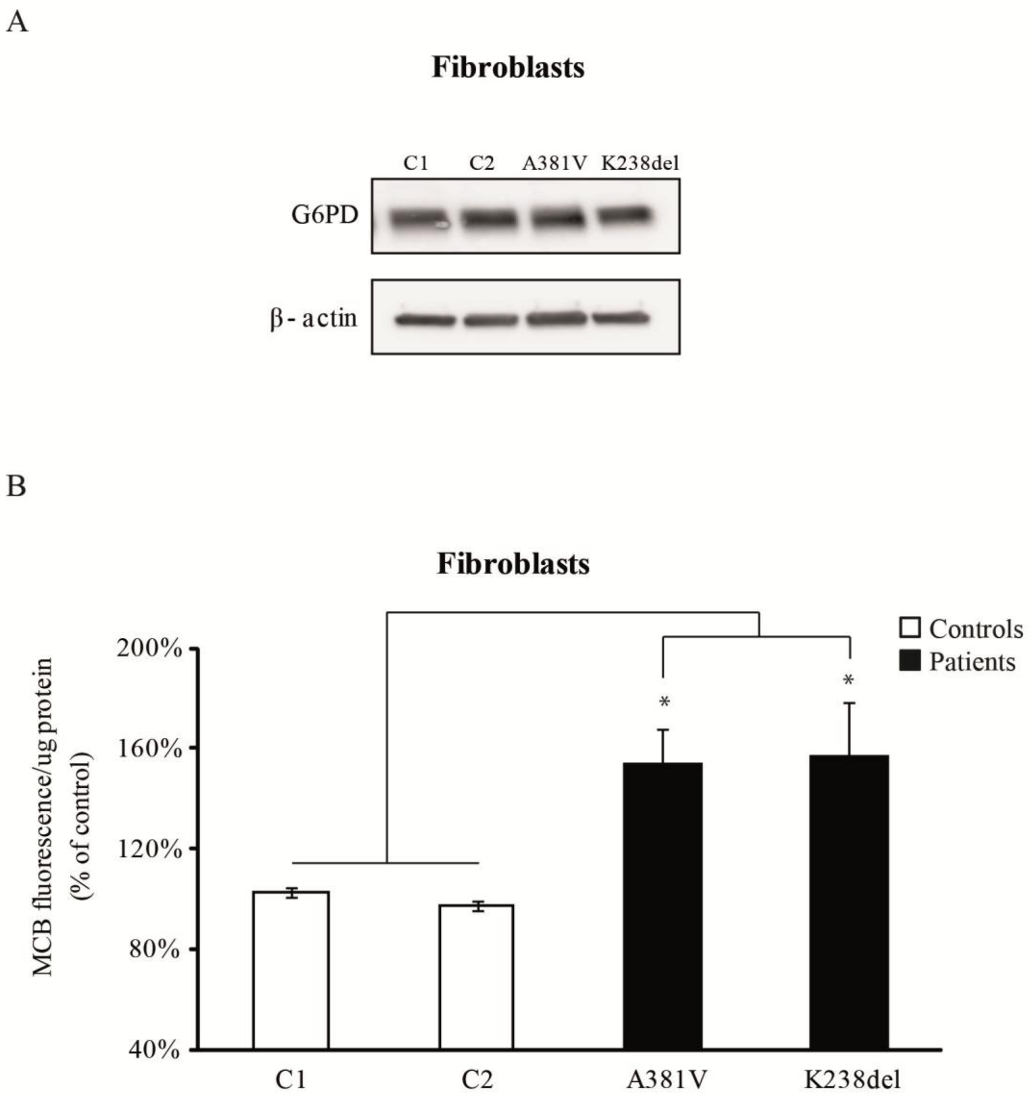

Supplementary Figure 2 – Glucose 6 phosphate dehydrogenase levels (G6PD) and GSH measurement related to the protein content in p62 mutation carriers compared to healthy controls. A – Immunoblotting showing glucose 6 phosphate dehydrogenase (G6PD) protein levels corresponding to whole cell lysates from fibroblasts from patients carrying p62 mutations and aged-matched controls C1 and C2 (B). β-actin was used as loading control. B – Histogram representing the GSH levels in fibroblasts from carriers of the p62 mutations and healthy controls related to the protein content. The histogram represents the percentage of fluorescence values from patient fibroblasts compared to controls. All data represents the mean of 4 independent experiments carried out in triplicates ± SEM. In all cases * indicates $p < 0.05$ compared with the values in control cells.

A

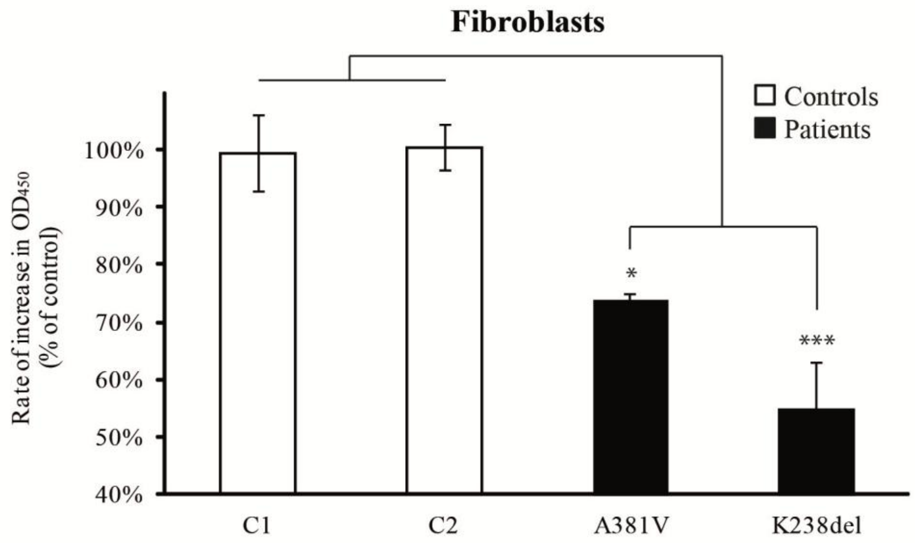

Supplementary Figure 3 – p62 deficient fibroblasts show inhibited complex I activity compared to healthy controls. A – Histogram representing the average complex I activity in fibroblasts from carriers of the p62 mutations and healthy controls. Complex I activity was measured by the rate of increase in absorbance OD450, normalized to control cells from three independent experiments. Data are represented as mean ± SEM. In all cases * indicates $P<0.05$ and *** indicates $P<0.001$ compared with the values in control cells.
